# Neonatal influenza infection causes pathological changes in the mouse brain

**DOI:** 10.1186/1297-9716-45-63

**Published:** 2014-06-10

**Authors:** Ji Eun Yu, Minsoo Kim, Jong-Hwan Lee, Byung-Joon Chang, Chang-Seon Song, Sang-Soep Nahm

**Affiliations:** 1 Laboratory of Veterinary Anatomy, College of Veterinary Medicine, Konkuk University, 120 Neungdongro, Gwangjingu, Seoul 143-729, Korea; 2 Avian Disease Laboratory, College of Veterinary Medicine, Konkuk University, 120 Neungdongro, Gwangjingu, Seoul 143-729, Korea

## Abstract

Influenza A virus infections have been proposed to be associated with a broad spectrum of central nervous system complications that range from acute encephalitis/encephalopathy to neuropsychiatric disorders in humans. In order to study early influenza virus exposure in the brain, we created an influenza-infection model in neonatal mice to investigate infection route and resulting pathological changes in the brain. Real-time polymerase chain reaction and immunohistochemical analyses showed that influenza virus infection induced by an intraperitoneal injection was first detected as early as 1 day post infection (dpi), and the peak infection was observed at 5 dpi. The viral antigen was detected in a wide range of brain regions, including: the cerebral cortex, hippocampus, cerebellum, and brainstem. Apoptotic cell death and gliosis were detected in the areas of viral infection. Significant increases in proinflammatory cytokine expression were also observed at 5 dpi. Viral RNAs were detected in the cerebrospinal fluid of infected adult mice as early as 1 dpi. In addition, many infected cells were observed near the ventricles, indicating that the virus may enter the brain parenchyma through the ventricles. These results demonstrate that influenza virus may effectively infect broad regions of the brain through the hematogenous route, potentially through the cerebrospinal fluid along the ventricles, and subsequently induce neuropathological changes in the neonatal mouse brain.

## Introduction

Highly pathogenic influenza A virus infection is a zoonosis that results in high mortality in both animals and humans. While influenza virus primarily targets the respiratory system, it is capable of infecting the central nervous system (CNS) in humans. Many clinical cases with neurological symptoms in the human population have been reported from 2009 influenza pandemic [1-4] and from recent highly pathogenic influenza virus (H5N1) infection cases [5-7].

Accumulating lines of evidence have shown that influenza virus infection can lead to diverse and prolonged neurological disturbances when the virus infects the nervous system. One of the most interesting findings is that a number of patients who recovered from influenza virus infection during pandemics such as the 1918 Spanish flu developed neuropsychiatric disorders, including: depression, schizophrenia, and Parkinsonism [8]. Moreover, a report has shown that influenza virus is detected in the substantia nigra in many patients who died from Parkinsonism [9], which provides a compelling link between influenza infection in the brain and the development of neurological disorders in humans. Recently, additional scientific evidence derived using animal models suggests a direct relationship between influenza virus infection and the occurrence of neurological disorders [10-12].

Several animal models have been used to study the pathogenesis that is induced by influenza virus infection [13,14]. Among them, mice and ferrets have been primarily used to study influenza-associated neurologic disease [13,15]. In addition, diverse inoculation methods, such as intranasal, intraperitoneal, and intracranial injections, have been employed to infect animals at different ages and for research purposes [16-18]. In this study, we used an intraperitoneal infection method in neonatal mice to mimic the hematogenous spread that results in systemic infection [17,19]. Neonatal mouse models have been previously used to study the impact of human infections during late second/early third trimester of gestation on human brain development [20].

Early exposure to influenza infection might cause persistent impairments in normal brain function [21-23]. Therefore, it is necessary to elucidate how influenza virus infects the nervous system at an early age and what pathological effects persist after infection. Previous reports using animal models have shown that the hippocampus, substantia nigra, prefrontal cortex, and cerebellum, as well as the paraventricular midline thalamic, medial habenular, and hypothalamic nuclei, are the brain regions that are affected after influenza virus infection [17-20]. Thus, we generated an influenza-infection model system in neonatal mice to study the neuropathological changes in the brain in response to early influenza virus exposure at this critical period.

Because elucidating the time course of infection and the routes of virus invasion into the brain is important for understanding the neuropathogenesis caused by influenza virus infection, several attempts have been made to investigate the possible routes of viral entry into the brain. To date, the suggested infection route of influenza virus is mostly via transsynaptic transport through cranial nerves, including the vagal and trigeminal nerves, in mice [10,24-26]. Some other hypotheses have suggested that the virus is disseminated through the blood circulation [26-28] or through the olfactory nerves [10,11]. In our study, we sought to decipher the neuropathological changes that were caused by neonatal influenza infection and to elucidate the infection route to the brain. To this end, the temporal and spatial distribution of influenza virus was examined, and the subsequent pathological changes were determined. Lastly, the potential route of influenza entry into the brain after intraperitoneal infection was investigated.

## Materials and methods

### Animal model and sample preparation

In order to determine the LD_50_ (lethal dose of 50%) of influenza A virus infection in neonatal mice, 5-day-old Balb/c mice with an average body weight of 2.6 g were randomly assigned to each group (*n* = 10). Mice were inoculated intraperitoneally with 30 μL of mouse-adapted neurotropic influenza A/NWS/33 virus (H1N1, ATCC VR-219) propagated in specific pathogen-free embryonated chicken eggs. Three concentrations of influenza A virus (10^2^, 10^3^, or 10^4^ TCID_50_/mL) were used. The mice were observed daily for 3 weeks to determine the mortality rate. LD_50_ was calculated by Miller and Tainter method with minor modifications [29].

Once the LD_50_ was determined, the mice were intraperitoneally infected with a dose of 30 μL of 10^3^ TCID_50_/mL virus. Five-day-old Balb/c mice were infected with the virus, sacrificed at 1, 2, 3, 4, 5, 7, 10, 15, and 21 days post infection (dpi), and then compared to age-matched control mice (*n* = 6/each dpi). The brains were collected from each mouse and cut along the midsagittal plane. Half of the brain sections were fixed in 10% neutral-buffered formalin, and the rest were immediately stored at -80 °C. The formalin-fixed brains were processed for routine paraffin embedding, and the frozen brains were used for RNA extraction.

The experimental protocols for the care and use of laboratory animals were approved by the Institutional Animal Care and Use Committee at Konkuk University (Permit No.: KU13040). Throughout the protocols, all efforts were made to minimize the number of animals used and their suffering. All mice were kept under a 12 h light: 12 h dark cycle. Food and water were given ad libitum up to the time of experiments. Animals were monitored daily after influenza infection until sample collection. During this study, humane endpoints were used in accordance with Konkuk University standard operating protocols. Mice that became cachectic or showed signs of paralysis were euthanized before sample collection. For each time point, mice were anesthetized with intraperitoneal injection of zoletil (90 mg/kg) and xylazine (10 mg/kg) and euthanized by cervical dislocation for sample collection according to standard protocols.

### Real-time polymerase chain reactions for the quantification of influenza A virus and proinflammatory cytokines

Total RNA was extracted from each mouse brain (*n* = 6/each dpi except *n* = 10 for 5 dpi) and from whole blood with TRIzol reagent (Gibco, Life Technologies Corporation, Grand Island, NY, USA) according to the manufacturer’s instructions. cDNA was synthesized from 4 μg of total RNA using oligo dT primers with a PrimeScript™ II 1^st^ strand cDNA Synthesis Kit (Takara Bio Inc., Otsu, Japan). The Viral RNA in the whole brain and blood was quantified by using M gene-based real-time polymerase chain reactions (PCR) with a TaqMan probe (QIAGEN Inc., Valencia, CA, USA), as previously described [30]. Quantification of proinflammatory cytokines (interleukin (IL)-1β, IL-6, and tumor necrosis factor (TNF)-α) in the whole brain was performed with a SYBR Premix Ex Taq™ II Kit (Takara Bio Inc.) in a CHROMO4™ (Bio-Rad Laboratories, Inc., Hercules, CA, USA) real-time PCR system according to the manufacturer’s instructions, with minor modifications. To control the variation in the DNA amount available for PCR, the expression of the target genes was normalized in relation to the expression of glyceraldehyde 3-phosphate dehydrogenase (GAPDH), which is an endogenous control. Each sample of the target and reference gene was run in duplicate in separate wells. The PCR cycling conditions included an initial step at 95 °C for 10 min, which was followed by 40 cycles of 95 °C for 30 s, 55 °C for 30 s, and 72 °C for 30 s, and a final step at 72 °C for 5 min. The 2 - ΔΔCT method was used to calculate the relative fold-changes in gene expression [31]. The specific primer sequences for the detection of influenza virus and proinflammatory cytokines are shown in Table 1.

**Table 1:** Sequence of primers and probe used in PCR

	**Sequence (5′ - 3′)**
**M**	F: AGA TGA GTC TTC TAA CCG AGG TCG
	R: TGC AAA AAC ATC TTC AAG TCT CTG
	P: FAM-TCA GGC CCC CTC AAA GCC GA-TAMRA
**HA**	F: GAA AGC TCA TGG CCC AAC CA
	R: TCC CAG GGG TGT TTG ACA CT
**IL-1β**	F: GAC CTT CCA GGA TGA GGA CA
	R: AGG CCA CAG GTA TTT TGT CG
**IL-6**	F: TAG TCC TTC CTA CCC CAA TTT CC
	R: CAG TGA GGA ATG TCC ACA AAC TG
**TNF-α**	F: CCG ATG GGT TGT ACC TTG TC
	R: CGG ACT CCG CAA AGT CTA AG
**GAPDH**	F: TTC ACC ATG GAG AAG GC
	R: GGC ATG GAC TGT GGT CAT GA

### Histological techniques and immunohistochemical staining procedures

To localize the infected virus in the brain (*n* = 3/each dpi), serial 4-μm-thick sections were made from the midsagittal plane, and every 20^th^ section was stained for influenza A virus. The sections were quenched with 0.3% H_2_O_2_ in methanol and subsequently blocked with 5% equine serum in phosphate-buffered saline (PBS) for 1 h at room temperature. The sections from control and infected mice brains were incubated with the H1N1 influenza A virus antibody (10 μg/mL, Abd Serotec, USA) overnight at 4 °C. The sections were then incubated with biotinylated anti-goat IgG (3.75 μg/mL, Vector Laboratories, Inc., Burlingame, CA, USA) for 2 h at room temperature and subsequently with horseradish peroxidase-conjugated streptavidin (Vector Laboratories, Inc.) for 1 h at room temperature. The virus was visualized using diaminobenzidine as a substrate and counterstained with methyl green. The stained slides were dehydrated, mounted, and evaluated with a light microscope (Olympus Corporation, Tokyo, Japan). Negative control staining was carried out by omitting primary antibody incubation. The locations of the infected virus were identified by positive signal, and the intensity of the signal was defined as follows: -, negative signal; +/-, weak signal; and +, positive signal [32].

To evaluate the pathological changes caused by the infection, brain sections were subjected to routine hematoxylin and eosin (H&E) staining. In order to detect glial cell activation and proliferation the sections were immunohistochemically stained with an antibody to glial fibrillary acidic protein (GFAP; 5.8 μg/mL, EMD Millipore Corporation, Billerica, MA, USA), which is a specific marker for astrocytes, and an antibody to ionized calcium-binding adapter protein 1 (iba1; 1 μg/mL, Wako Pure Chemical Industries, Ltd., Osaka, Japan), which is specific marker for the macrophage/microglia lineage. The slides were incubated with the appropriate secondary antibodies and horseradish peroxidase-conjugated streptavidin and then processed as described above.

Double-immunofluorescence staining was also carried out to identify the types of cells that were infected with influenza A virus. Deparaffinized brain sections were stained for the H1N1 influenza A virus antibody (10 μg/mL, Abd Serotec) and subsequently incubated with a mouse monoclonal antibody to neuronal nuclei (10 μg/mL, EMD Millipore Corporation), a rabbit polyclonal antibody to GFAP (5.8 μg/mL, EMD Millipore Corporation), or a rabbit antibody to Iba1 (1 μg/mL, Wako Pure Chemical Industries, Ltd.) for 4 h at 4 °C. Then, the sections were incubated with a fluorescent dye conjugated with anti-mouse IgG (DyLight™ 488, Thermo Fisher Scientific Inc., Waltham, MA, USA) or an anti-rabbit IgG (DyLight™ 405, Thermo Fisher Scientific Inc.) for 2 h at room temperature. Subsequently, the slides were mounted with Vectashield (Vector Laboratories, Inc.) and examined under an Olympus BX-51 fluorescence microscope (Olympus Corporation).

### Analysis of cell death

A terminal deoxynucleotidyl transferase (TdT) dUTP nick-end labeling (TUNEL) assay was performed in deparaffinized tissue sections with an In Situ Cell Death Detection Kit (Roche Diagnostics GmbH, Mannheim, Germany) according to the manufacturer’s protocols. Antigen retrieval was performed by heating the sections in a microwave oven in 10 mM sodium citrate buffer, pH 6.0, for 4 min. Thereafter, the sections were incubated with TdT and fluorescein-labeled dNTP in Tris–HCl buffer (30 mmol/L, pH 7.2) at 37 °C for 60 min. Incorporated fluorescein was detected by an anti-fluorescein antibody conjugated with horseradish peroxidase for 30 min at 37 °C. The slides were then incubated with diaminobenzidine for the signal conversion and counterstained with methyl green. A negative control was performed by omitting TdT, and mouse prostatic tissue was used as a positive control for the TUNEL reaction.

### Confirmation of apoptotic cell death

In order to determine if influenza infection evoked apoptotic cell death, influenza-infected primary brain cell culture was treated with a pan-caspase inhibitor. The primary brain cell culture was prepared from dissected mouse brain at postnatal day 5 with a Papain Dissociation System (Worthington Biochemical Corporation, Lakewood, NJ, USA). All procedures were conducted according to the manufacturer’s instructions with minor modifications. Dissected cerebral cortex, brainstem, and cerebellum were immediately incubated in a papain (20 U/mL) digestion solution containing DNase I (0.005%, v/v) at 37 °C for 15 min. Then, the cells were dissociated by gentle pipetting and subsequently centrifuged for 5 min at 200 × *g*. The dissociation process was inhibited by an ovomucoid protease inhibitor with bovine serum albumin and DNase I and finally centrifuged for 6 min at 100 × *g*. The cells were resuspended and plated on a laminin-coated 6-well plate in Dulbecco’s Modified Eagle’s Medium (DMEM) containing 10% fetal bovine serum (FBS, Life Technologies Corporation) and penicillin-streptomycin (100 units/mL; Sigma-Aldrich Co. LLC, St. Louis, MO, USA). Cultured cells were maintained at 37 °C in a humidified atmosphere with a 5% CO_2_ supplement.

The primary cultured cells were seeded in a 96-well plate (10^5^ cells/well) with 10% (v/v) FBS in DMEM. The cultured cells were inoculated with influenza virus at a dose of 10^7^ TCID_50_/mL. After a 40-min incubation, Z-VAD-FMK (20 μM, Promega Corporation, Madison, WI, USA) was added with additional media to each well in the pan-caspase-inhibitor-treatment group. To determine the effects of the pan-caspase inhibitor on cell viability after influenza infection, a neutral red uptake assay was performed according to the protocols previously described [33]. Cell viability was measured by a neutral red uptake assay at 12 h after infection. Neutral red (40 μg/mL) was added to each well, and the cells were incubated for 2 h at 37 °C. Then, the neutral red medium was removed, and each well was washed with PBS. Finally, the neutral red absorbed by viable cells was extracted by Sorenson’s citrate buffer to measure the total amount of neutral red at 540 nm.

### CSF analysis in infected mouse brain

The cerebrospinal fluid (CSF) from infected mice was collected according to the method of Liu and Duff [34]. Six-week-old Balb/c mice were used instead of 5–10-day-old mice due to technical difficulties associated with collecting adequate amount of CSF from neonates. The mice were infected with influenza virus intraperitoneally at a dose of 10^7^ TCID_50_/mL. CSF samples from at least 3 mice were taken at 1, 2, 3, 4, and 5 dpi. The infected mice were anesthetized with avertin and placed prone on the stereotaxic instrument. A midsagittal incision of the skin was made inferior to the occiput. Under the dissection microscope, the subcutaneous tissue and neck muscles through the midline were bluntly separated with microforceps. Then, the mouse was laid down so that the body made an approximately 135° angle with the head. At this angle, the dura of the cisterna magna was visible with a glistening reverse triangle, and a blood vessel could be seen. The dura was carefully penetrated with a pulled glass capillary (Borosilicate glass, B100-75-10, World Precision Instruments, Inc., Sarasota, FL, USA) that had a tapered tip with an inner diameter of 0.5 mm. Following a perceptible change in the resistance to the capillary insertion, the CSF was collected into the capillary. The average volume of the CSF obtained was approximately 2–4 μL. All samples were stored at -80 °C until the PCR analysis. Conventional PCR was employed to amplify virus from CSF directly due to extremely limited amount of CSF collected from mouse brain. Repeated conventional PCR of the CSF was performed with the primers specific for influenza virus HA genes under the same conditions. The amplified product, which was expected to yield a 500-bp sequence, was analyzed by 1.5% agarose gel electrophoresis. Further nucleotide sequencing was performed by ABI Prism system (ABI 3730xl, Life Technologies Corporation).

### Statistics

All statistical analyses were performed with GraphPad Prism version 5.0 (GraphPad Software, Inc., La Jolla, CA, USA). The measured data were given as median except mean ± standard error of the mean (SEM) for cell viability tests in vitro. The differences in the cytokine analyses between the control and infection groups were examined statistically with Mann–Whitney tests. Kruskal–Wallis tests, which were followed by Dunn’s multiple comparison tests, were used for both the cell viability tests in vitro and virus quantification with real-time PCR in the brain. Differences with *p* values less than 0.05 were considered significant.

## Results

### Determination of LD_50_ of influenza virus in neonatal mice

**Figure 1:**
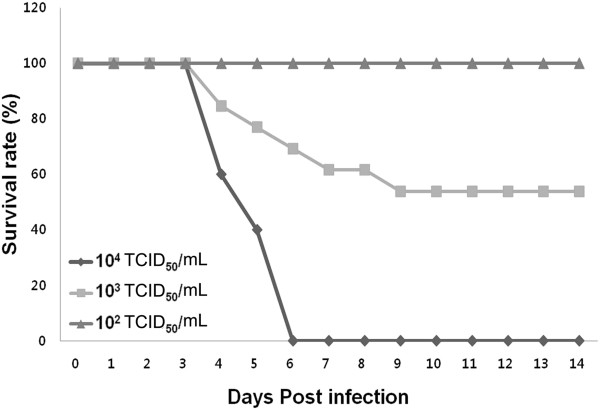
**Dose-dependent survival rates of intraperitoneal influenza virus infection in 5-day-old mice.** Mice were infected with increasing doses of the A/NWS/33 virus, and their survival rates were observed for 3 weeks. The LD_50_ of the A/NWS/33 virus in neonatal mice was determined to be 10^3^ TCID_50_/mL by intraperitoneal injection (*n* = 10/group).

The influenza-infected mice were carefully observed daily for their clinical symptoms and body weight changes for 3 weeks. While no mice died in the 10^2^-TCID_50_/mL group, the infected mice started to die from 4 dpi in the 10^3^- and 10^4^-TCID_50_/mL groups. In particular, mice that were infected with 10^4^-TCID_50_/mL of the influenza virus showed 100% mortality within 6 dpi. However, the mice infected with 10^3^-TCID_50_/mL of the influenza virus continued to die until 9 dpi and showed a 50% survival rate after 9 dpi. Thus, the LD_50_ of the A/NWS/33 virus in neonatal mice was determined to be 10^3^ TCID_50_/mL by intraperitoneal injection. Mortality from influenza infection at a dose of LD_50_ occurs primarily between 5 dpi and 6 dpi (Figure 1).

### Influenza virus detection and localization in the infected brain

**Figure 2:**
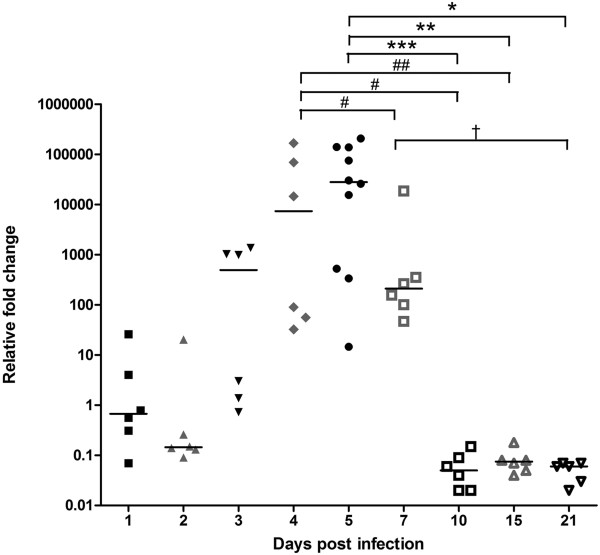
**Influenza virus quantification in the infected brain.** The quantitative real-time polymerase chain reaction (PCR) results showed influenza virus infection as early as 1 dpi and a peak infection at 5 dpi. Viral infection was detected until 21 dpi, but only weak signal was detected from 10 dpi to 21 dpi. Data were expressed as median (*n* = 6/dpi except *n* = 10 for 5 dpi; #, *p* < 0.05 or ##, *p* < 0.01 at 4 dpi; *, *p* < 0.05 or **, *p* < 0.01 or ***, *p* < 0.001 at 5 dpi; †, *p* < 0.05 at 7 dpi).

To determine if the intraperitoneally infected influenza virus could enter the brain, we tried to detect influenza virus using PCR as well as immunohistochemistry. We first conducted real-time PCR to confirm the presence of the influenza virus in the brain and the peak infection time. The real-time PCR results showed that the influenza virus infection took place as early as 1 dpi, and the peak infection was observed at 5 dpi (Figure 2). At 5 dpi, there was a significant increase in the viral M gene levels compared to those at the other sampling time points. No viral M gene was detected in all blood samples tested.

**Figure 3:**
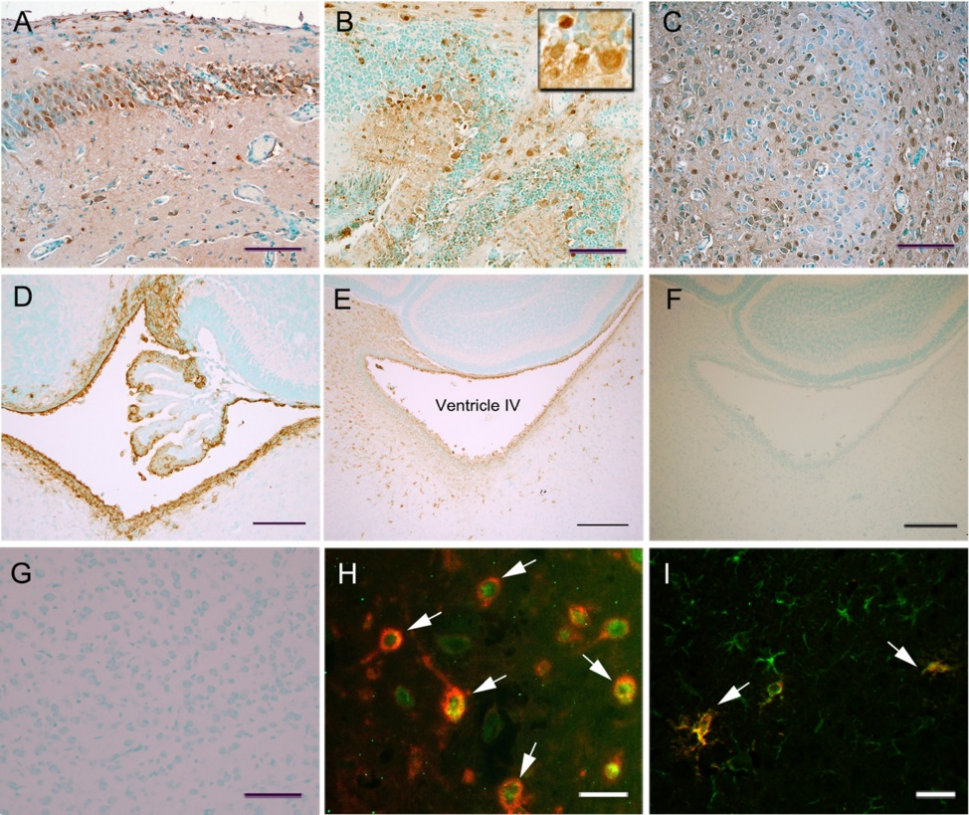
**Immunohistochemical detection of intraperitoneally infected influenza virus in the brain.** Influenza virus was detected in the hippocampus **(A)**, the cerebellum (**B**, inset shows infected Purkinje cells), the thalamus **(C)**, choroid plexus of fourth ventricle **(D)**, and the periventricular region **(E)** of infected mouse brain at 5 dpi. No viral antigen was detected in the infected mouse brain stained without primary antibody **(F)** and the thalamus of SPF mouse brain **(G)**. Double-immunofluorescence staining for influenza virus (red, **H-I**) and neuronal marker (NeuN, green, **H**) or astrocyte marker (GFAP, green, **I**) show either virus-infected neurons or astrocytes. Scale bar for **A-D**, **G** = 100 μm; for **E-F** = 200 μm; for **H-I** = 25 μm.

The real-time PCR and conventional PCR results showed that the intraperitoneally infected influenza virus could enter the brain. In order to confirm PCR results and rule out blood contamination of the brain, we further tried to localize influenza virus infected within the brain parenchyma by using immunohistochemical techniques. The immunohistochemical detection of influenza virus showed that viral infection in the brain occurred as early as 1 dpi and that the virus was detected until 5 dpi (Table 2). Similar to the peak in the PCR results, the peak viral infection was observed at 5 dpi. At 5 dpi, many virus-infected cells were observed in multiple brain regions, including i) the dentate gyrus, subiculum, postsubiculum, and CA1 region of the hippocampal formation, ii) Purkinje neurons, granule cells, and neurons of the deep cerebellar nuclei in the cerebellum, iii) reticular nucleus in the midbrain, iv) the caudoputamen and globus pallidus, v) the olfactory bulb, anterior cingulate area, prelimbic area, somatomotor area, motor area, and retrosplenial area of the cerebral cortex, vi) the pontine reticular nucleus and tegmental reticular nucleus in the pons, vii) the dorsal thalamus in the diencephalon, viii) the nucleus raphe magnus and nucleus raphe obscurus in the medulla oblongata and ix) ventricular ependymal cells, as well as choroid plexus epithelial cells (Figure 3, Table 2). It should be noted that the viral antigen that was detected near the periventricular region exhibited a unique pattern with a gradual increase. In this area, more infected cells were detected close to the ventricle, while less infected cells were observed away from the ventricle (Figure 3E). Control staining was carried out to validate specificity of the antibody against influenza virus by omitting primary antibody for staining infected mouse brain sections or in specific pathogen free (SPF) mouse brain sections. No viral antigen was detected in infected brains stained without primary antibody or SPF mouse brains (Figure 3F and 3G).To determine the types of infected cells, double-immunofluorescence staining was performed either with influenza virus and a neuronal marker (NeuN) or with influenza virus and an astrocyte marker (GFAP). The results revealed that influenza virus could infect both neurons and astrocytes (Figure 3H and 3I).

**Table 2:** Immunohistochemical distribution of viral antigen in infected mouse brain

Dpi*	No.**	Localization of viral antigens***						
		**Cerebral cortex**	**Hippocampus**	**Diencephalon**	**Midbrain**	**Pons**	**Medulla oblongata**	**Cerebellum**
**1**	**1**	**-**	**-**	**-**	**+**	**+**	**+**	**-**
	**2**	**-**	**-**	**+**	**+**	**-**	**+**	**-**
	**3**	**-**	**-**	**-**	**+**	**+**	**+**	**-**
**2**	**1**	**+/-**	**-**	**-**	**-**	**-**	**-**	**+/-**
	**2**	**+/-**	**-**	**-**	**-**	**-**	**+**	**+/-**
	**3**	**-**	**-**	**-**	**-**	**+**	**+**	**+/-**
**3**	**1**	**-**	**-**	**-**	**-**	**-**	**+**	**+**
	**2**	**-**	**-**	**-**	**-**	**-**	**+/-**	**+/-**
	**3**	**-**	**-**	**-**	**-**	**-**	**+/-**	**+/-**
**4**	**1**	**-**	**-**	**-**	**-**	**-**	**-**	**+/-**
	**2**	**+**	**-**	**+**	**-**	**-**	**+**	**+/-**
	**3**	**+**	**-**	**+**	**+**	**+**	**+**	**+**
**5**	**1**	**+**	**+**	**+**	**+**	**-**	**-**	**-**
	**2**	**+**	**-**	**+**	**+**	**+**	**+**	**+**
	**3**	**+**	**-**	**+**	**+**	**+**	**+**	**+**
**7**	**1**	**-**	**-**	**-**	**-**	**-**	**-**	**-**
	**2**	**-**	**-**	**-**	**-**	**-**	**-**	**-**
	**3**	**-**	**-**	**-**	**-**	**-**	**-**	**-**

The temporal and spatial localization of virus was evaluated by the presence and intensity. No viral antigen was detected at 10, 15, and 21 dpi (data not shown). (*: Days post infection; **: *n* = 3/days post-infection; ***: -, negative signal; +/-. weak signal; +, positive signal).

### Histopathological assessment of the infected brain

**Figure 4:**
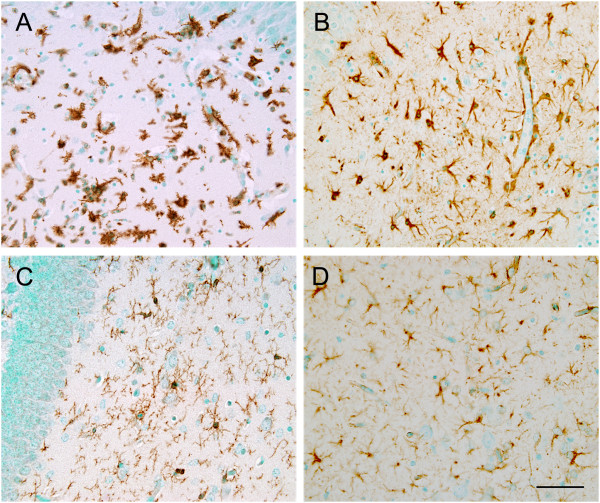
**Glial activation of the influenza-infected brain.** Immunohistochemical staining of microglia **(A, C)** and astrocytes **(B, D)** in the hippocampus of infected mice at 5 dpi with age-matched control mice. Note that many glial cells with activated morphology are seen in the infected mice brain **(A, B)**, whereas resting glial cells are stained in control mouse brain **(C, D)**. Scale bar = 50 μm.

In order to investigate the histopathological changes in the influenza-infected brain, H&E-stained sections were examined at 1, 2, 3, 4, 5, 7, 10, 15, and 21 dpi. Surprisingly, we were not able to detect any dramatic histopathological changes in most of the sections examined. The only recognizable findings were mild parenchymal neutrophil infiltration and neuronophagia in the cerebral cortex at 5 dpi (data not shown). However, subsequent immunohistochemical staining for microglia and astrocytes showed that both types of glial cells were markedly activated and had proliferated, particularly at 5 dpi, when compared to control mouse brain (Figure 4A and 4D).

### Apoptotic cell death caused by neonatal influenza infection

**Figure 5:**
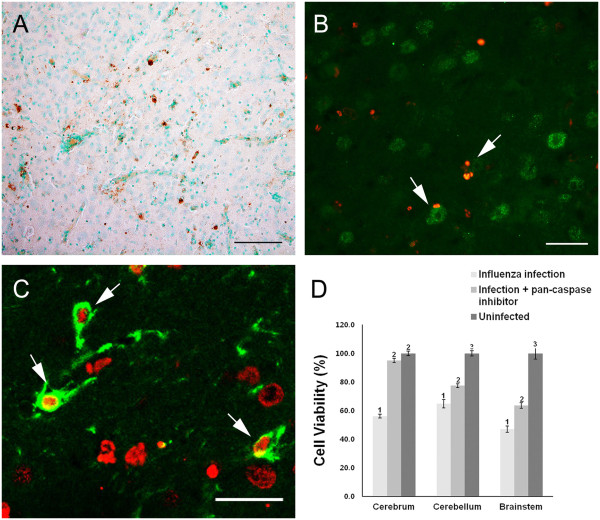
**Apoptotic cell death caused by neonatal influenza infection. (A)** A representative image of a terminal deoxynucleotidyl transferase dUTP nick-end labeling (TUNEL) assay in virus-infected brain shows many TUNEL-positive cells in the thalamus, which indicates cells degenerating by apoptosis. Double-immunofluorescence staining for TUNEL (Red, **B**-**C**) and the neuronal marker (NeuN, green, **B**) or astrocyte marker (GFAP, green, **C**) show either neurons or astrocytes undergoing apoptotic cell death (arrows in **B**, **C**). Scale bar for **A** = 100 μm; for **B** = 50 μm; for C = 25 μm. **(D)** Comparison of cell viability with pan-caspase inhibitor treatment in influenza-infected primary brain cell culture. Primary cultured neuronal cells (10^5^ cells/well) were treated with a Z-VAD-FMK inhibitor (20 μM) after virus inoculation. Statistical analysis of the three groups shows a significant increase in cell viability with pan-caspase inhibitor treatment. The different numbers above the bars indicate significant statistical difference (Dunn’s multiple comparison test, mean ± SEM, *p* < 0.0001).

As the H&E-stained brain sections showed the presence of some cells undergoing degenerative changes, we sought to determine the mode of cell death that was caused by the influenza infection. A TUNEL assay in the infected brain revealed that many of the cells were undergoing apoptotic cell death in various brain regions at 5 dpi (Figure 5A). To confirm that influenza infection caused apoptotic cell death, a pan-caspase inhibitor was added to the primary neuron/glia-mixed culture simultaneously with influenza virus inoculation in vitro. The pan-caspase inhibitor treatment resulted in a significant increase in viable cells (Figure 5D). In particular, the viability of the cells that were prepared from the cerebral cortex reached nearly 95% with the pan-caspase inhibitor treatment. In contrast, the viability of the cells prepared from the cerebellum and brainstem was increased, but it was still significantly lower than the viability of the control culture. Double-immunofluorescence staining showed that both neurons and astrocytes were infected by influenza inoculation and that both types of cells underwent apoptotic cell death (Figure 5B and 5C).

### Changes in proinflammatory cytokine expression in the infected brain

**Figure 6:**
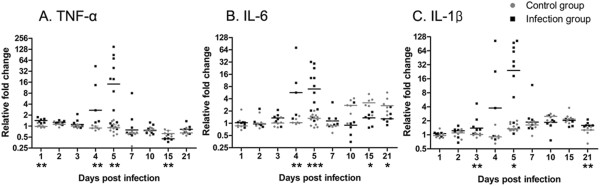
**Real-time polymerase chain reaction analysis of proinflammatory cytokine expression in the influenza-infected mouse brain.** All data were normalized against the levels of glyceraldehyde 3-phosphate dehydrogenase (GAPDH) expression. The levels of tumor necrosis factor (TNF)-α **(A)**, interleukin (IL)-6 **(B)**, and IL-1β **(C)** all show a similar temporal change in that mRNA expression at 5 dpi was higher than that in control brain. Data were expressed as median (n = 6/dpi except n = 10 for 5 dpi; *, *p* < 0.05; **, *p* < 0.01; ***, *p* < 0.001).

To assess the immune responses of the brain against influenza virus infection, changes in proinflammatory cytokines, which are known to induce inflammation, were investigated. The temporal changes in TNF-α, IL-6, and IL-1β mRNA expression were analyzed by quantitative real-time PCR at 1, 2, 3, 4, 5, 7, 10, 15, and 21 dpi. The mRNA levels of TNF-α, IL-6, and IL-1β showed a marked increase at 5 dpi, and they then returned to the baseline levels at 10 dpi. There was a similar temporal trend in the expression of the 3 cytokines in the infected brain, and all cytokines showed a statistically significant difference at 5 dpi (Figure 6).

### Influenza virus detection in the CSF from infected brain

**Figure 7:**
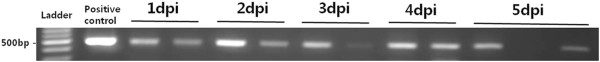
**Detection of virus RNA in the cerebrospinal fluid (CSF) of infected mice.** A representative image of a gel electrophoresis analysis of the HA gene sequence that was amplified from the CSF. The virus was detected in the CSF of mice from 1 dpi to 5 dpi.

One of the most important findings of the distribution of the virus in the brain was the gradual increase in the occurrence of virus-infected cells near the ventricle (Figure 3E). This finding suggested that the virus may enter the brain through the ventricle. Thus, we tried to detect the virus in the CSF of infected mice. PCR analysis showed the presence of influenza virus as early as 1 dpi, and it continued to be detected until 5 dpi (Figure 7). The final amplified PCR products were further validated by sequencing. Sequence comparisons of the amplified product and the HA gene confirmed that influenza was present in the CSF. This result indicated that the CSF might contribute to influenza infection in the brain.

## Discussion

To the best of our knowledge, observation of pathological changes of influenza-infected neonatal mouse in time-dependent manner has not been reported. Our study showed that intraperitoneally injected influenza virus could enter the neonatal mouse brain and induced brain cell death, which eventually resulted in neuroinflammation. The value of our study is the demonstration of the temporal and spatial localization of influenza virus in the brain caused by intraperitoneal infection and to provide potential infection routes of influenza virus in the brain. Although neuropathogenesis has been well described for intranasal influenza inoculation in adult animals [12,24,25,35], few studies have been carried out to investigate neuropathological changes after neonatal infection. Therefore, we chose to use an intraperitoneal inoculation method with mouse-adapted neurotropic influenza A virus (A/NWS/33) to elucidate the neuropathologies caused by neonatal infection.

A previous study has reported the temporal localization of influenza virus after intranasal inoculation [10,36]. Park et al. [10] reported that the intranasally inoculated highly pathogenic avian influenza virus was observed in the nasal mucosa beginning at 1 dpi. According to their findings, the viral antigens were first detected in the brainstem at 4 dpi, and it was not until 5 dpi that viral antigens became widely distributed throughout a variety of brain regions. In addition, Majde et al. [36] have demonstrated that mouse-adapted non-neurotropic human influenza viral antigens were detected in mouse olfactory bulb within the first 15 h after intranasal infection. Although their results and our findings were not directly comparable because the viral virulence and infection methods were different, it is quite interesting to note that the rate of viral spread in the brain was quite comparable regardless of the differences in viral virulence and infection routes.

In our study, the virus-infected brain regions were the hippocampus, the cerebellum, the brainstem, the striatum, the cerebral cortex, as well as the periventricular regions including the ventricular ependymal cells and choroid plexus epithelial cells, which were in part coincident with the distributions of the virus in the mouse brain that have been described [10,25]. Iwasaki et al. [25] have described virus-infected brain areas, which were consistent with the findings of our studies. However, Park et al. [10] and Matsuda et al. [37] have reported that virus-infected cells were observed in the nucleus of the solitary tract and nucleus ambiguous in the brainstem. We were not able to detect influenza virus in those areas. On the other hand, highly pathogenic influenza virus was identified in the cerebral cortex, the brainstem, the cerebellar Purkinje cells, the internal capsule, and the adjacent thalamus in ferret brains [38]. In particular, Yamada et al. [35] have described that influenza virus was observed in the ependymal cells lining the ventricles and in the choroid plexus epithelial cells, but not in any of the endothelial cells of the blood vessels in the ferret brain, which was in accordance with our findings. Such discrepancies in the virus distribution from various studies may be attributable to the animal species, the virus strains, and the inoculation routes that are used in the studies.

We found that many neurons and astrocytes had degenerated via apoptosis in the virus-infected regions, and this may result in permanent brain dysfunction if the infection occurred during the period of brain maturation. Apoptosis is known to be involved in various neurological disorders, including virus-induced CNS diseases [39]. It has been reported that influenza virus induces apoptotic neuronal death in the anterior olfactory nucleus of mice [40] and cultured primary cells [41]. Interestingly, we observed that the increase in cell viability induced by the pan-caspase inhibitor treatment was different across brain areas. In particular, the cell death caused by influenza infection in the cerebellum and brainstem was not inhibited to the same degree as in the cerebrum. These results suggest that other cell death mechanisms might contribute to cell death in these brain regions. However, no direct evidence of other cell death pathways has been presented until now. In addition, we observed severe gliosis, which was possibly due to neuronal death in the infected brain. These findings support previous reports suggesting that influenza A virus induces severe neuroinflammation in the brain accompanied by gliosis [24,42,43].

There have been a number of previous studies on the infection routes of influenza virus into the CNS after intranasal inoculation. Currently, it has been proposed that influenza virus reaches the CNS primarily though the cranial nerves [10,24,37,44] or the peripheral nerves [10]. It has also been suggested that the olfactory pathway is an additional minor infection route to the brain in mice [10], as well as a major infection route to the brain in infected ferrets [11,35]. Furthermore, hematogenous spread by breakdown of the blood–brain barrier (BBB) has been suggested as the infection route of influenza virus to the brain [45]. Virus invasion through hematogenous routes implies that the virus could cross either the BBB in the vascular endothelium of the brain or the blood-CSF barrier that is restricted to the epithelial cells of the choroid plexus in the ventricular system [46].

Based on our results, influenza infection through the cranial nerves alone does not seem to fully explain the virus distribution in the neonatal mouse brain because the brain lesions caused by influenza virus were widely distributed throughout the brain, and the virus antigen was not prominently stained in the nuclei that are associated with cranial nerves. Previous studies have shown that the levels of proinflammatory cytokines including TNF-α, IL-6, and IL-1β are increased at day 6 or 7 after influenza A virus infection [42,45]. In addition, it has been suggested that an influenza infection-induced cytokine storm could lead to vascular hyperpermeability [45]. Therefore, we initially considered that a hematogenous spread caused by BBB dysfunction might be an additional infection route to the brain. Similar to the findings of previous studies, our data showed that proinflammatory cytokine expression was markedly increased near 5 dpi. Interestingly, IL-6 expression levels of control brains appeared to be higher than those of infected brains at 15 dpi and 21 dpi. The reasons for this phenomenon are still unclear, because little information is available for changes in IL-6 expression at this age in control mice. It has been suggested that the presence of influenza virus receptors and the detection of viral antigens in endothelial cells in the brain parenchyma could support hematogenous viral spread to the brain [27,28]. However, we did not observe that endothelial cells were infected with influenza virus, while no viral RNA was detected in blood by real-time PCR in our study (data not shown).

The possibility of influenza virus entry into the brain via CSF by crossing the blood-CSF barrier has been suggested in ferrets [35] and chickens [28]. They have reported that the virus was found in CSF from infected animals. Either ependymal cells in the ventricle or choroid plexus epithelial cells were positive for viral antigen [28,35]. Although we detected viral RNA in the CSF using adult mice instead of using neonates, it is possible that CSF plays important roles in influenza infection to the brain of neonatal mice. In addition, there are some clinical cases that influenza virus was detected in CSF in human cases of influenza-associated encephalitis/encephalopathy [5,47-49].

Yet there has been no direct evidence of influenza virus entry into the brain through the CSF in mice. Thus, we tried to detect influenza virus in the CSF of infected mice to show that influenza virus could enter the brain through the ventricles. The BBB begins to form between E11 - E17 in the mouse and tight junctions are present at that period. Thus, the movement of macromolecules is restricted [50,51], indicating the BBB permeability of neonates seems to be similar to that of adult mice. We found that the virus RNA was detected in the CSF of infected adult mice from 1 dpi to 5 dpi, and this was in accordance with virus detection near the periventricular region and ventricular ependymal cells. Proving the routes of influenza virus infection into the brain was the most challenging experiment in our study. Taken together, virus localization near the ventricle and the presence of viral RNA in the CSF provide quite persuasive evidence that the ventricles and CSF are important conduits of influenza virus to the brain.

## Competing interests

The authors declare that they have no competing interests.

## Authors’ contributions

JY conceived, designed the study, carried out most of the experiments, analyzed data and prepared the manuscript. MK, JL and BC performed histological and statistical analysis. CS carried out virus preparation. SN conceived, designed, coordinated the study, analyzed data and manuscript preparation. All authors read and approved the final manuscript.
